# Short-course intensification with enfuvirtide in virologic failure: impact on intracellular HIV reservoir and on viral tropism (INNOVE study)

**DOI:** 10.1186/1758-2652-13-S4-P40

**Published:** 2010-11-08

**Authors:** J Ghosn, C Delaugerre, A Pinta, N Pierre, D Etienne, F Raffi, L Morand-Joubert

**Affiliations:** 1 AP-HP, Bicetre University Hospital, Le Kremlin Bicetre, France; 2 AP-HP, Saint Louis University Hospital, Paris, France; 3 Roche France, Neuilly sur Seine, France; 4 Nantes University Hospital, Nantes, France; 5 AP-HP, Saint Antoine University Hospital, Paris, France

## Background

Overall, studies of ART intensification in suppressed patients showed no reduction in HIV reservoir, as assessed by proviral DNA. Limited data about impact of ART intensification are available for failing patients harbouring multidrug resistant viruses. We studied the effects of intensification with enfuvirtide (ENF) on intracellular HIV reservoir and tropism evolution under treatment selective pressure in this clinical setting.

## Methods

Innove is a prospective, open-label multicenter study in pretreated patients harboring viruses still susceptible to at least 2 active compounds on genotypic resistance test performed at pre-inclusion. Patients with confirmed virologic failure were randomized to optimized regimen (OBR) plus 12-week short-course intensification with ENF n=14 or only OBR n= 15. Genotypic tropism and quantification of HIV-1 intracellular-DNA were determined in total peripheral blood mononuclear cells (PBMC) obtained at baseline, W4, W12 and W24. HIV intracellular-DNA was quantified by a real-time PCR assay (Biocentrics). V3 env sequences were amplified from PBMC HIV-1 DNA and interpreted according to Geno2pheno (10% false positivity).

## Results

At inclusion, median plasma HIV-RNA was 4 log10 cp/mL, median CD4 346 cells/mm3 and patients harbored viruses with a median of 4 INTI-associated resistance mutations (RM), 1 INNTI-associated RM and 9 PI-associated RM. Median HIV-DNA (log10/106 PBMC) was 5.05 at baseline, 4.91 at W24 (4.96 log in OBR and 4.81 log in ENF+OBR). The decrease being not significant overall nor between groups: median change from baseline to W4 was -0.19 and -0.19 and to W24; -0.09 and -0.29 in OBR and ENF+OBR groups, respectively . At baseline, predominant variants were DM/X4 in 10 patients and R5 in 17 patients. At W24, tropism switches were observed in 6 patients : 1 from DM/X4 to R5 and 5 from R5 to DM/X4. Among these 5 patients with R5-X4 switch, plasma HIV-RNA was <50 cp/ml at W24 in 5/5, none received maraviroc, and 4/5 were randomised in ENF+OBR group.

## Conclusion

In patients with therapeutic failure and harboring resistant viruses with a GSS≥2, a 12-week short-course intensification with enfuvirtide did not yield any impact on intracellular reservoir. In this intracellular compartment, R5-X4 switch can occur despite achievement of maximal virologic suppression with enfuvirtide-containing regimen.

